# The 100 Most Influential Papers and Recent Trends in the Field of Gastrointestinal Stromal Tumours: A Bibliometric Analysis

**DOI:** 10.7759/cureus.2311

**Published:** 2018-03-12

**Authors:** Khadijah Siddiq, Hira F Akbar, Meeshal Khan, Amna A Siddiqui, Salman Nusrat, Jean Y Blay,

**Affiliations:** 1 Civil Hospital Karachi, Dow University of Health Sciences (DUHS), Karachi, Pakistan; 2 Gasteroenterology, University of Oklahoma Health Sciences Center, Oklahoma City, United States; 3 Centre Léon Bérard, Lyon, France.

**Keywords:** gastrointestinal stromal tumors, gists, bibliometric analysis, scopus, citescore

## Abstract

Background

Bibliometric analysis is a statistical tool used to examine the exponential growth in medical research. Many analogous analyses have been conducted, but none existed for gastrointestinal stromal tumors (GISTs). Hence, we conducted a citation analysis of the hundred most cited and recently published articles on this topic.

Methods

Scopus was chosen as the primary database, through which the top 100 and recent publications were ranked according to the citation count and were then analysed.

Results

The 100 most cited articles were published between 1992 and 2013, among which the greatest number of articles were published in the years 2002 (n = 15) and 2006 (n = 11). Amidst the 24 countries from which the articles originated, the United States of America (n = 76) topped the list. The Journal of Clinical Oncology (n = 15) and the American Journal of Clinical Pathology (n = 10) contributed majority of the top articles. Harvard Medical School alone produced 44 of the top 100. Articles from 2013 to date showed the same trend as that of top 100 articles regarding origin and institutions.

Conclusion

Basic science and genetics of GISTs are established, and new drugs are being studied for medicinal therapy. Surgical management and diagnostics of these tumors, however, are yet to be studied as extensively.

## Introduction

Explosive growth in the medical literature has contributed to massive leaps within evidence-based medicine, which combines clinical expertise of a physician with current scientific research to provide adequate patient care [1,2].

All this growth in the academic arena requires a method by which researchers can efficiently track the most impactful advances and identify the pressing challenges. Bibliometric analysis, a statistical tool by which frequency and trends of citations of the published literature undergo quantitative scrutiny, can fulfil this objective [3,4]. Medical research is historically a competitive field and bibliometrics can serve as a guide to examine research performance from a global perspective, pinpointing successful advances and breakthroughs of individual countries, researchers, and journals [5]. Several such analyses have been conducted for various topics, such as breast cancer [6], orthopaedic surgery [7], epilepsy [8], thrombolytic therapy [9], and valvular heart diseases [10]. Within oncology, however, our thorough search indicated that there has been no bibliometric analysis of the literature on gastrointestinal stromal tumors (GISTs).

Although infrequent, GISTs are the most common mesenchymal tumors of the gastrointestinal tract [11], with an incidence at 10–15 per million per year [12]. Introduced as a diagnostic term in 1983 [13], GISTs have emerged from being poorly defined, treatment-resistant tumors to treatable tumor entities used as a paradigmatic cancer model for multidisciplinary, targeted therapy directed against a driver oncogene [14-16]. Citation Classics [17] on GISTs are also recognized in our article, providing an insight into the specific aspects the scientific community appears to be focusing on, the existing gaps and possible directions for future global research [8].

The present bibliometric analysis accumulates all relevant data represented by the 100 most cited articles on GIST. This work will enable researchers to acquire the latest information about the work being done in this arena, while identifying future challenges to focus on.

## Materials and methods

A citation search was conducted to identify the topmost 100 cited articles in the available literature, as well as top 50 articles from 2013 to the current date, concerning gastrointestinal stromal tumors. Coequal to other researchers [9,10], we chose Elsevier’s Scopus online database (http://www.scopus.com) for our bibliometric analysis, as it provides 20% more coverage than Web of Science with more accurate citation counts than Google Scholar [18]. However, full articles were accessed from PubMed, Excerpta Medica dataBASE (EMBASE), and Science Direct.

The keywords ‘Gastrointestinal Stromal Tumors (GIST)’, ‘Gastrointestinal Pacemaker Cell Tumors (GIPACT)’ [19], and ‘Gastrointestinal Sub-epithelial Tumors’ were obtained from Medical Subject Headings (MESH) of PubMed and sections (C49.A0-5) and (C49.A9) of the International Classification of Diseases 10 (ICD-10). All electronic database searches were performed on August 22 and 23, 2017. Keywords were searched in ‘article titles’, ‘abstracts’, and ‘keywords’. Relevant articles were retrieved and sorted by the option of ‘Cited by’, which gave us the articles arranged in descending order of their number of citations. No filters of language, time, human studies, subject area, territory, or affiliations were used. Abstracts and full texts of the articles were read from the sorted list and irrelevant ones were removed. GISTs are soft tissue tumors with mesenchymal origin [20], but studies regarding soft tissue tumors that did not primarily discuss GISTs were excluded.

All article types excluding those requiring manual searching, telephone access, guidelines, and non-PubMed indexed articles, were included. The dataset was further evaluated, examining title, first and senior author, institution, department of the first author, topic, source, year of publication, and country of origin. In contrast with other researchers [9,10], we used CiteScore [21], Source Normalized Impact per Paper (SNIP) and SCImago Journal Rank (SJR), to rank our journals. Some articles were cited more frequently than others due to differences in time since publication. We eliminated this error by determining citation index for each article.

Citation analysis of the articles extracted was conducted both on Scopus and by manual screening of the articles. They were classified into three broad categories: Basic Sciences, Therapeutic, and Diagnostic. Tables and charts were created using Microsoft Excel 2016. IBM Statistical Package for the Social Sciences (IBM SPSS Statistics for Windows, Version 20.0. Armonk, New York) was used to apply the Pearson product moment correlation co-efficient to evaluate the relationship between citation times, CiteScore and citation density. The Mann-Whitney U test was applied to determine whether there was any significant difference in citations of review and original articles. The Kruskal-Wallis test was used to evaluate any significant difference between subject area and year, and also between distributions of citations among the subject area. p-value < 0.05 was considered significant in all cases.

## Results

Top 100 article trends

(a) Citation Count, Citations per Year and Citation Trend

**Figure 1:**
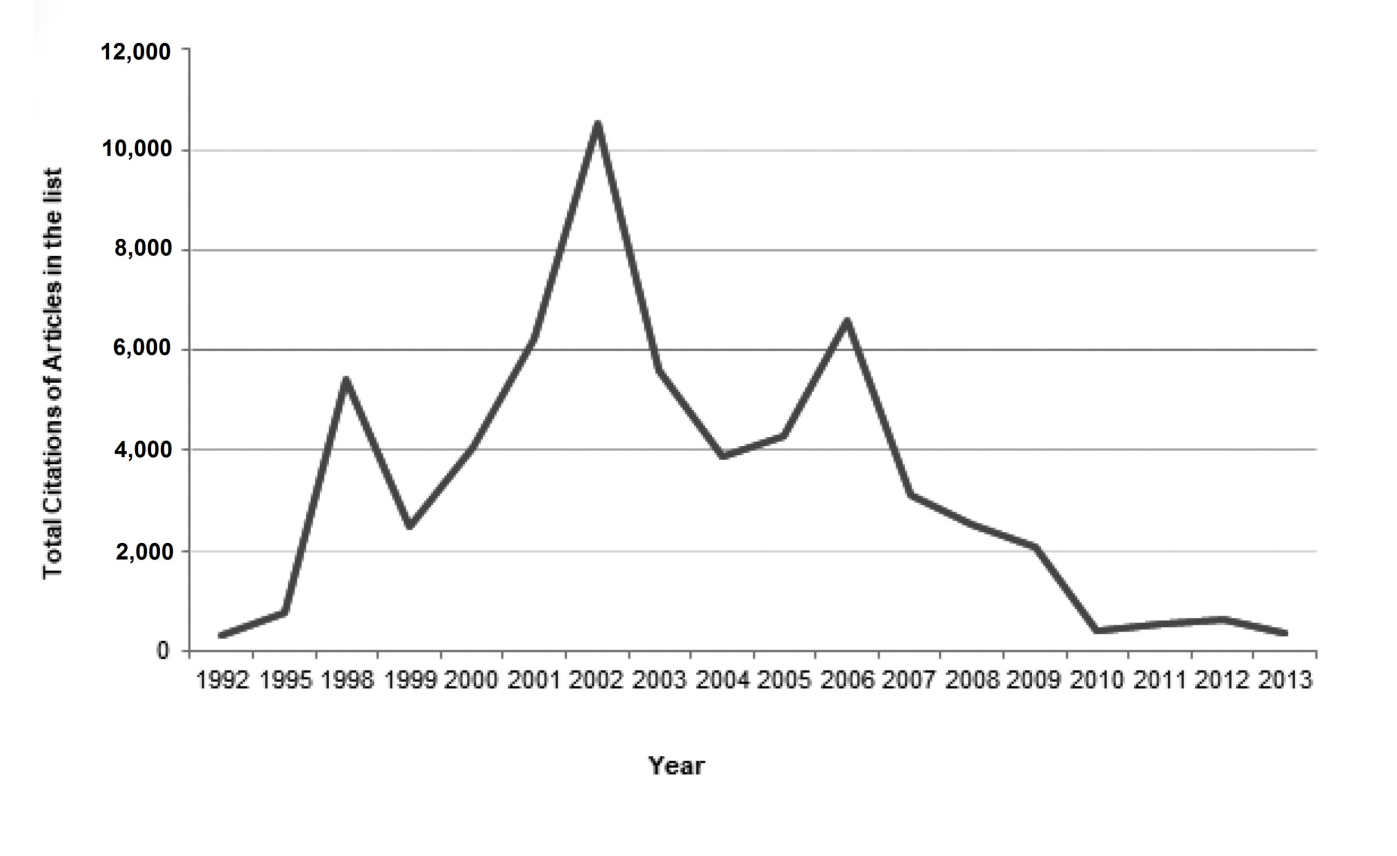
Citations per year.

The top 100 articles were published between 1992 and 2013 (see Appendix A). Most were original articles (n = 79), while others comprised review articles (n = 19), conference papers (n = 1), and letters to the editor (n = 1). The citations of those articles summed up to 59,911, ranging from 260 to 3,076, with a median of 396 and a mean of 599.11 (interquartile range 281). Approximately 7% were self-cited and 4% were book citations, reducing the original citations to 53,520. Citation density (citations per year) ranged from 12.12 to 203.13, with a median of 32.18 and a mean of 47.29 (interquartile range 38.02). A significant positive correlation was found between citation and citation density (r = 0.883) and citation and CiteScore (r = 0.223) but there was no significant correlation between citation and year of publication. Figure 1 shows the trend of total citations by year.

(b) Origins, Institutions, and Authorships

**Figure 2:**
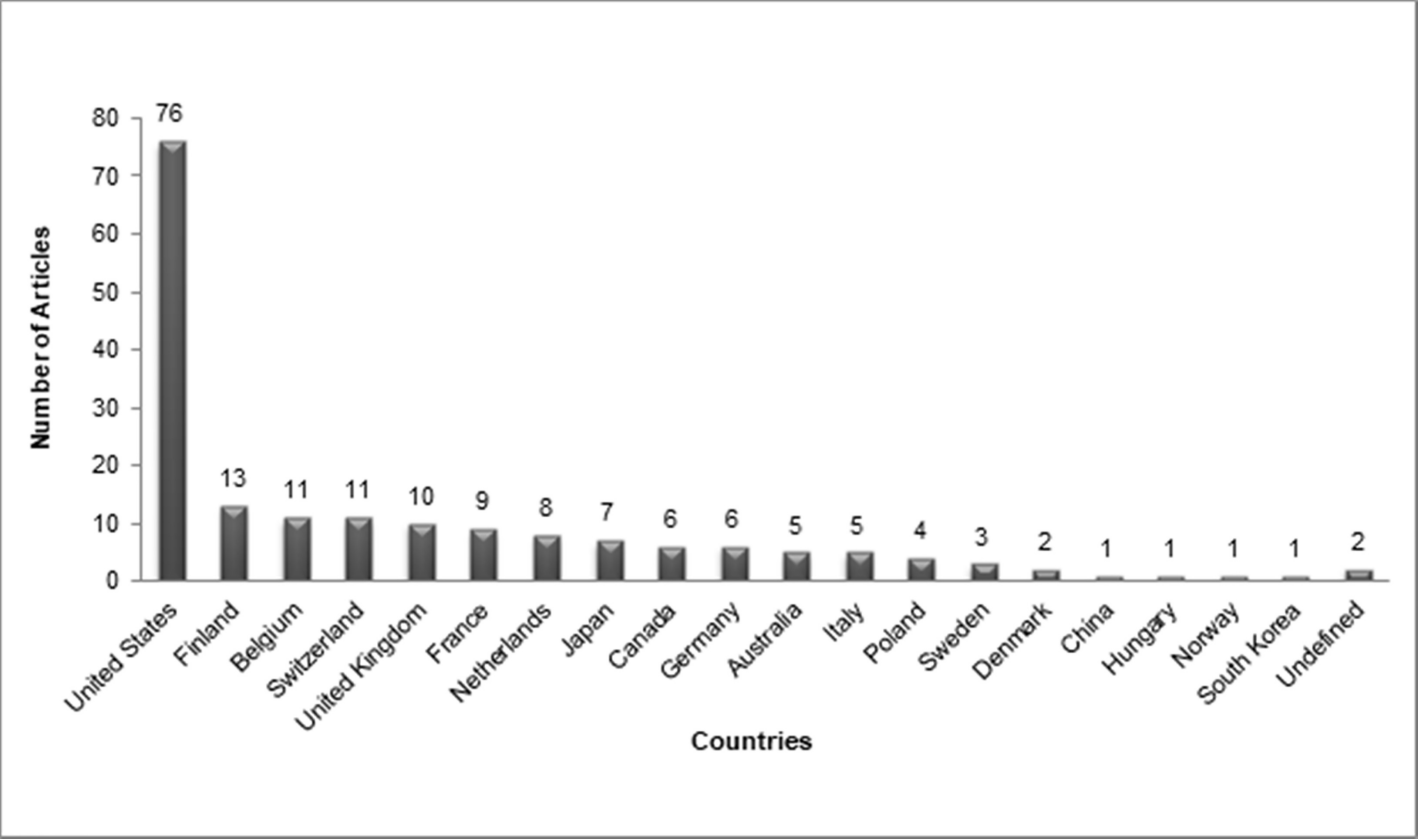
Articles originating from each country.

The top 100 articles were produced by 24 different countries, with almost half (n = 44) of the articles having contributions from more than one country. The USA (n = 76), Finland (n = 15), and Belgium (n = 12) were the top contributors (Figure 2).

In addition, 202 different institutions were associated with the top 100 articles. Harvard Medical School (n = 44) and University of Helsinki (n = 29) alone contributed to 73% of our top 100 articles on GISTs. Table 1 lists the top 10 institutions with eight or more articles among the top 100.

**Table 1:** Institutions with eight or more articles among the top 100.

Institutions	Number of Documents
Harvard Medical School	44
University of Helsinki	29
Armed Forces Institution of Pathology	18
Oregon Health and Science University	14
Memorial Sloan-Kettering Cancer Center	10
Novartis International AG	9
Royal Marsden Hospital, London	8
Erasmus University Medical Center	8
VA Medical Center	8
KU Leuven–University Hospital Leuven	8

A broad range of 528 authors contributed to the top 100 articles. Each paper had a median of eight authors. The number of authors per article ranged from 1 to 59. Authors with more than 10 articles in the list are shown in Table 2.

**Table 2:** Authors with more than 10 Articles in the top 100.

Author	Number of Articles	Author Position				Author Affiliations	H Index	Primary Topic of Interest	Years	Highest Citation
		First	Last	Others	Corresponding					
Demetri, GD	21	5	5	11	2	Dana-Farber Cancer Institution, Boston, United States	91	Medicinal Therapy and Genetics	2000-2013	3,050
Heinrich, MC	20	7	4	8	9	Oregon Health and Science University, Portland, United States	73	Medicinal Therapy and Genetics	2002-2011	3,050
Miettinen, M	19	14	4	1	14	National Cancer Institution, Laboratory of Pathology, Bethesda, United States	96	Pathology and Genetics	1995-2006	2,279
Fletcher, CDM	18	1	2	15	1	Brigham and Women's Hospital, Department of Pathology, Boston, United States	111	Medicinal Therapy and Genetics	2000-2008	3,050
Corless, CL	18	4	1	13	1	Oregon Health and Science University, Portland, United States	79	Medicinal Therapy and Genetics	2002-2011	3,050
Fletcher, JA	15	0	7	8	2	Brigham and Women's Hospital, Department of Pathology, Boston, United States	91	Medicinal Therapy and Genetics	2000-2011	3,050
Lasota, J	14	2	11	1	0	National Cancer Institution, Laboratory of Pathology, Bethesda, United States	56	Pathology	1999-2006	2,279
Joensuu, H	12	5	2	5	5	Helsinki University Central Hospital, Department of Oncology, Helsinki, Finland	87	Medicinal Therapy and Genetics	2001-2013	3,050
Von Mehren, M	12	0	0	12	0	Fox Chase Cancer Center, Philadelphia, United States	46	Medicinal Therapy	2002-2013	3,050
Sarlomo-Rikala, M	11	1	1	9	0	Helsingin Yliopisto, Department of Pathology, Helsinki, Finland	39	Pathology	1998-2012	1,603
Sobin, LHH	11	0	2	9	0	National Cancer Institution, Frederick National Laboratory for Cancer Research, Bethesda, United States	78	Pathology	1999-2016	2,279

(c) Journals

The top 100 articles were published in 40 journals, with half of the articles published in six journals. Table 3 shows these six journals, various analytical parameters, and their subject areas.

**Table 3:** Journals with more than five articles in the top 100. SNIP: Source Normalized Impact per Paper. SJR: SCImago Journal Rank

Journal Name	CiteScore	Highest CiteScore Percentile	CiteScore Rank	%Cited	SNIP	SJR	Subject Area	Number of Articles	Total Citations
Journal Of Clinical Oncology	10.11	98	6/321	71	4.89	8.883	Oncology	15	8,548
American Journal Of Surgical Pathology	5.05	99	2/372	87	2.36	2.371	Surgery	10	4,145
Lancet	6.93	99	20/2,156	43	13.7	12.47	Medicine	7	5,713
European Journal Of Cancer	6.1	95	15/321	87	2.16	3.011	Oncology	7	2,659
American Journal Of Pathology	4.1	94	11/182	84	1.19	2.209	Medicine	6	3,305
Human Pathology	2.84	88	21/182	78	1.13	1.302	Medicine	6	4,713

*(d) Subject Area and Topics* 

The top 100 GIST articles fell within three major subject areas: basic science, therapeutic, and diagnostic. More than half (n = 58) examined basic GIST science, while the remainder contributed to therapeutic (n = 40) and diagnostic (n = 10) knowledge about GIST. These major subject areas were further classified according to subtopics, as shown in Table 4. 7% of the articles had overlap between subject areas.

**Table 4:** Major subjects and sub-topics of the top 100 articles. *Numbers may not add up to 100. Numbers within each group may not add up to the total in each group, due to overlapping topics/sub-topics in the individual manuscripts.

Major Topics	Sub-topic	*Number of Articles
Basic Sciences	Genetic	24
	Pathology	20
	Prognosis	13
	Epidemiology	1
Therapeutic	Medical	37
	Surgical	2
	Both	1
Diagnostic	Radiological	7
	Others	2

**Figure 3:**
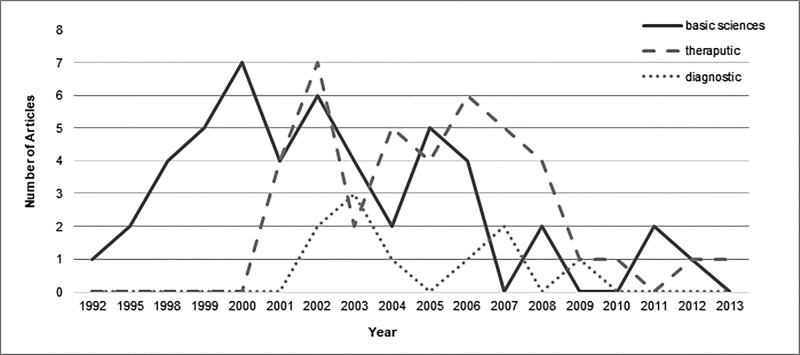
Number of articles of major subjects from top 100 articles published per year.

**Figure 4:**
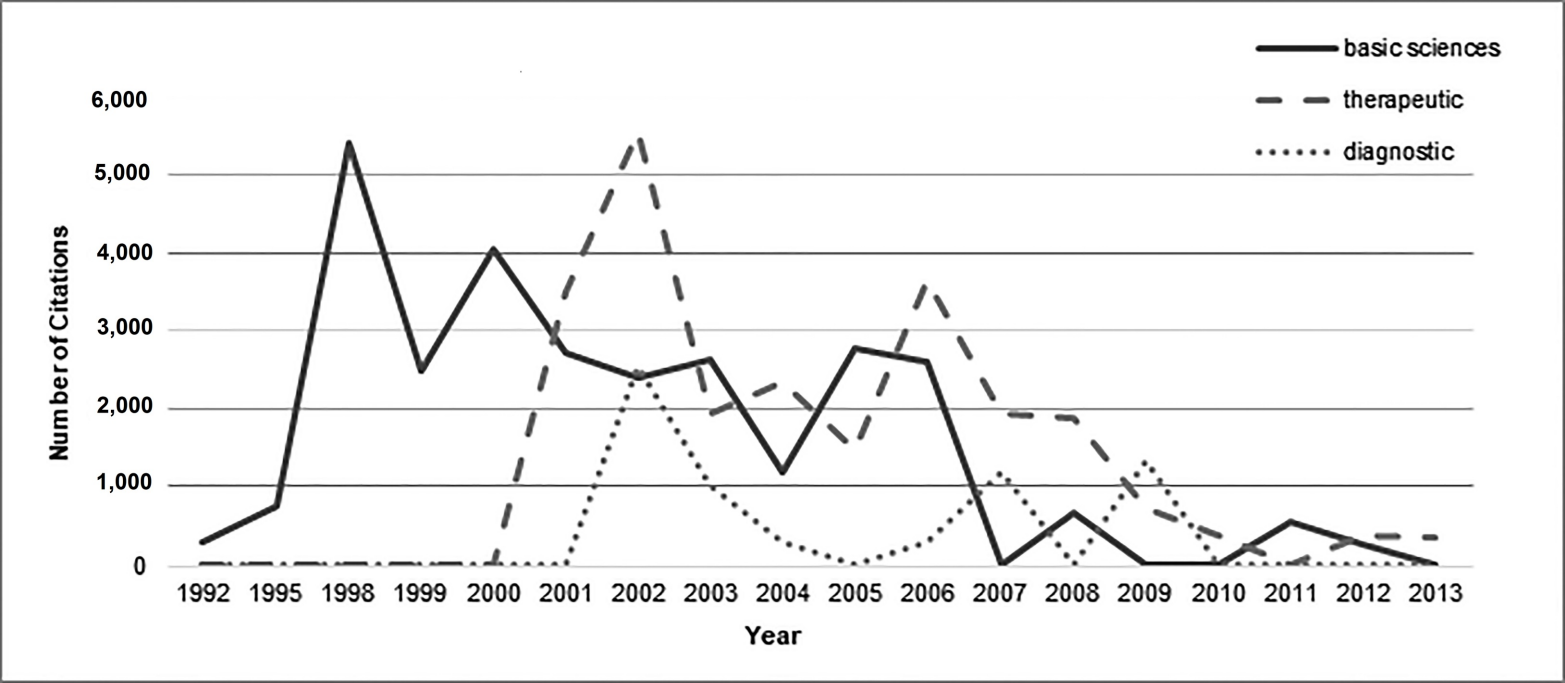
Number of citations of major subjects of top 100 articles per year.

A significant difference was found between the year of publication of articles and categories of subject areas (p = 0.00) but the distribution of citations was the same across subject area categories (p = 0.708). Figure 3 shows the trend of articles in different subject areas over time. Few articles imparting therapeutic or diagnostic knowledge about GIST were published before the year 2000. Most of the work in all three subject areas was performed from 2000-2010. Figure 4 displays the number of citations per subject area over time. As shown, the most citations for basic science articles were from 1992-2000. From 2000 on, citation counts were homogenous for all three subject areas.

Overall and to date trends

**Figure 5:**
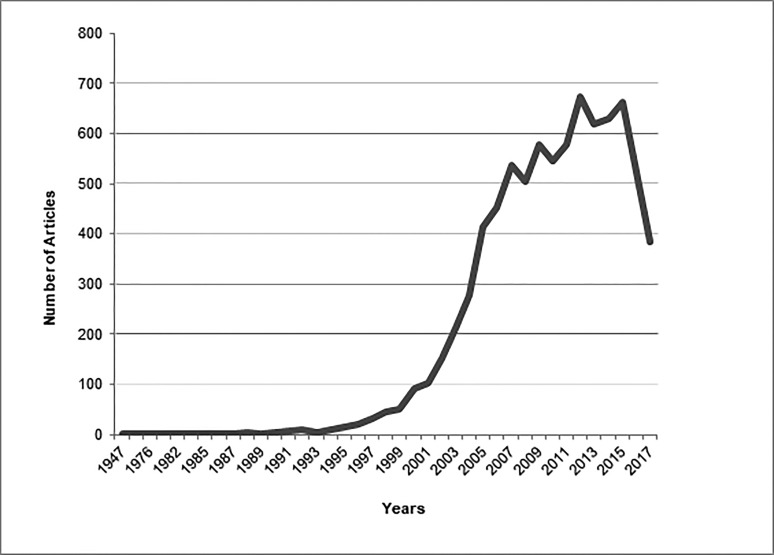
Overall trend of gastrointestinal stromal tumors (GISTs) articles.

Figure 5 shows the overall trend of GIST articles from 1947 to date, showing 2012 (n = 653) as the year in which most of the work was done on GISTs, followed by 2015 (n = 630). The analysis of top 50 cited articles from each year ranging from 2013 till now showed that the most contributing countries were the USA and Italy with 54.45% and 21.8% articles, respectively. Harvard Medical School was again on the top of the list with 40.09% articles. However, basic sciences, therapeutic and diagnostic studies constitute 46.03%, 42.07% and 11.88% of past five year’s top-cited articles, respectively. Demetri, GD (n = 13), Bauer, S (n = 11) and Blay, JY (n = 11) were the authors who worked eminently on the topic of GISTs in past five years.

## Discussion

The oldest article on GISTs, “Gant and his operations”, was published in 1947 and was not accessible through any online database. The second oldest relevant research paper accessible via Web of Knowledge, “Solitary solid stromal gastrointestinal tumors in von Recklinghausen's disease with minimal smooth muscle differentiation”, was published in 1984 and had 82 citations to date. Both these pioneer articles on GISTs did not make a place in the top 100 articles, hence indicating that the quality of the work plays a greater role in citation number than does the number of years the article has been a part of the literature.

Top 100 article citations and trends

(a) Citation Count, Citations per Year and Citation Trend

Among the top 100 articles, the eighty most frequently cited articles were published from 2000-2008. Throughout this nine-year span, more than five articles of top 100 list were published per year. The most-often cited article in the list was published in 1998, while the oldest of the top 100 articles cited was published in 1992. In accordance with previous studies [10], our graph of the total article citations over time (Figure 1) showed two peaks, the highest in 2002 (n = 15) and the second-highest in 2006 (n = 11). After 2006, a gradual decrease in citations occurred, followed by a rapid decrease in 2008. Five articles that were published after 2008 made to the top 100 list. This finding strengthens the idea that some topics undergo intense study at a certain time during which extensive research is performed, and after which the topic ceases to be of broad and current interest [22]. There was no significant difference between the citations of original research articles and review articles (p = 0.310), contradicting the belief that review articles are more often cited [10]. Interestingly, the second article on the list had the highest citation density.

(b) Countries of Origin, Institutions and Authors

A total of 76 of the top 100 articles came from the USA (Figure 2). Campbell explains this major contribution from the USA by stating that reviewers and authors from the USA show bias towards local papers [23]. Finland produced the second-highest number of quality papers (n = 13), followed by Belgium and Switzerland (both, n = 11). Only one paper from China was included in the top 100, despite GIST being most prevalent in that region [12].

Total 40 institutions contributed to our top 100 articles, all with at least two articles. Table 1 shows the top 10 institutions, four of which belong to the USA. A total of 55 of the extracted articles had multi-institutional origins. Of these, 25 papers had multinational origins, suggesting that international collaborations produce high-quality output that greatly benefits the scientific community [10].

None of the authors of the first article of top 100 articles were in the list of top 11 authors extracted, whereas seven authors of the second most-cited articles were in that list (Table 2). Each of the top 11 authors contributed to at least 11 articles. Fletcher, CDM had the highest H-index, but he ranked fourth in our list, as he had 18 articles among the top 100. Authors with a high H-index not only have a greater chance of having their work accepted, but are also more likely to get promotions and become reviewers [24].

(c) Journals

As previously mentioned, the top 100 articles were published in 40 journals. A total of 12 were oncology journals (41% of articles) and 16 were medicine journals (36% of articles). Among the oncology-based journals, Cancer Cell had the highest CiteScore of 16.19, followed by Nature Reviews Cancer, with a CiteScore of 15.79, but Journal of Clinical Oncology (n = 15) and The European Journal of Cancer (n = 7) has the most articles published in them (Table 3). Both the top CiteScore journals had only one article each in the top 100. Among the medicine-based journals, The New England Journal of Medicine had the highest CiteScore of 12.82 followed by the Lancet. The most cited article of the top 100 list was published in Science, a multidisciplinary journal with a CiteScore of 14.39. Only two articles published in Science appeared in the top 100 list. Nature Genetics had the highest CiteScore (20.83) overall, but only one article of top 100 was published in it. We observed a weak positive correlation of CiteScore with citation (r = 0.233).

According to the Bradford law these 28 journals, oncology and medicine-based summed up, may be considered our core journals [25]. This trend indicates that high-quality articles are published in field-specific journals, as also reported by other bibliometric analysis [18]. We used multiple analytical parameters to rank our journals, including CiteScore, SJR, and SNIP to reduce bias [26]. CiteScore is a metric similar to a journal’s Impact Factor that gives us a comprehensive view of the journal’s effect on the Scientific Community.

(d) Subject Areas

A total of 58 of the top 100 articles concerned basic sciences. Under the sphere of the basic sciences, 20 articles covered the pathological, histochemical, and immunological aspects of GISTs. Twenty-three articles discussed genetics, while 12 studied disease prognosis and three focused on epidemiology. Basic sciences publications were highest in 2000 (n = 7), while therapeutic publications were highest in 2002 (n = 7; Figure 3). However, papers from the basic sciences field that were most often cited were published in 1998 and there were more than 5,000 citations for both of these fields in these years as shown in Figure 4.

Thirty-four therapeutic articles focused on the medical treatment of GISTs with imatinib, sunitinib, tyrosine kinase inhibitors, or other chemotherapeutic drugs. The articles examined treatment efficacy, mechanisms of action, associations, reactions, adverse effects, prognosis, and mechanisms of resistance. The remaining six articles examined surgical management or a combination of surgical and medicinal treatment. The most-cited articles from the therapeutic field were published in 2000. Only 10 of the top 100 articles pertained to the field of diagnostics. Most work imparting therapeutic or diagnostic information was published between 2000 and 2010; none was published before 2000 (Figure 3). Several articles targeted both a genetic basis and medical therapy for GISTs; hence, we categorized these articles as both therapeutic and diagnostic. Similarly, prognosis and surgical management were discussed simultaneously in some articles.

Overall and to date trends

**Figure 6:**
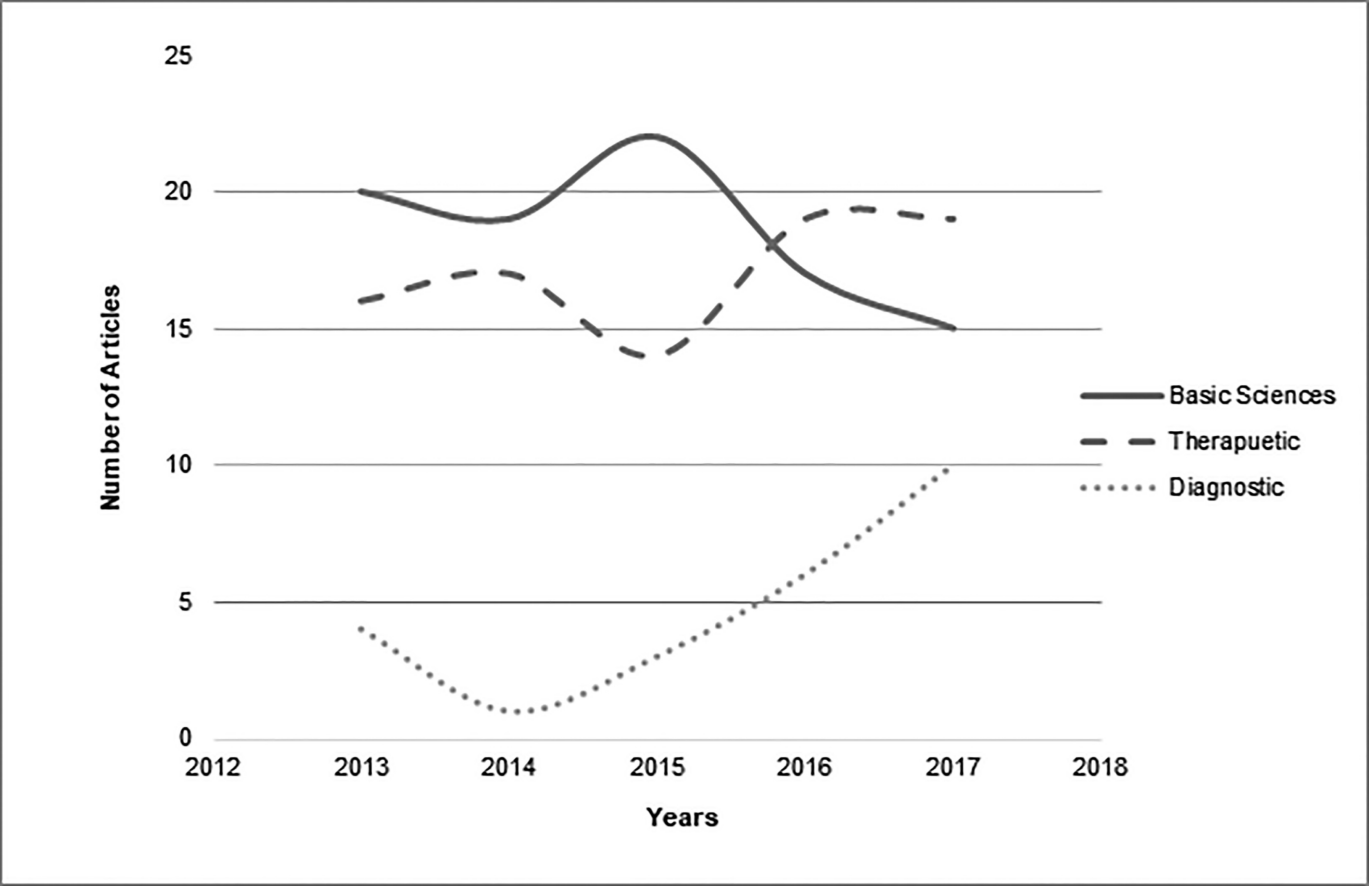
Number of articles in each subject area published from 2013 to date.

**Figure 7:**
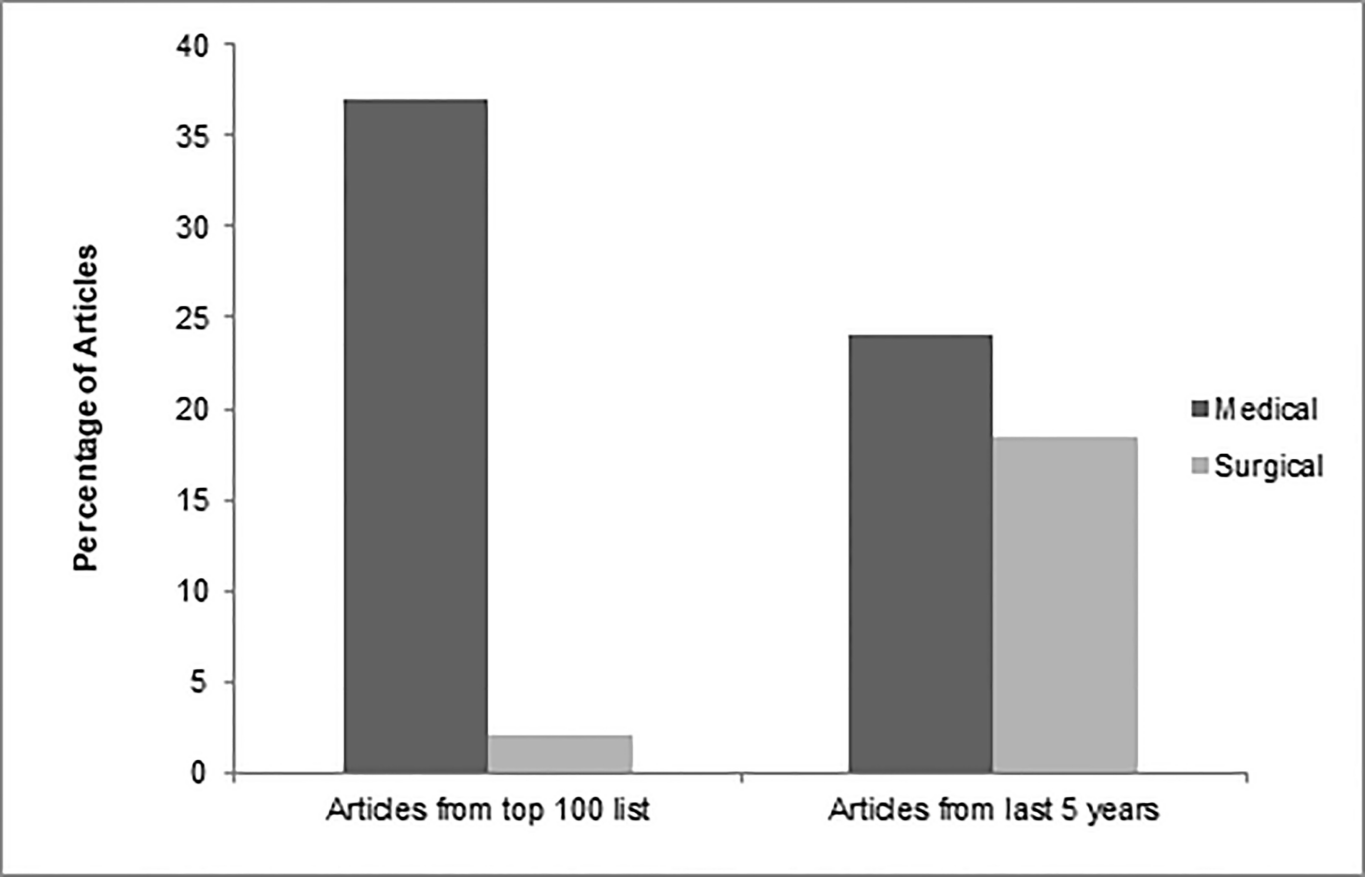
Percentages of therapeutic articles published.

Our extensive recent analysis showed us that in contrast to other topics that are ‘hot’ in some era and got most of the work done on them in a specific period of time  [22], GISTs was a progressive topic. Despite the fact that most cited articles on GISTs were from the last decade, the current decade is the one in which most of the work has been conducted (Figure 5). Moreover, contrary to the top 100 articles on GISTs, a considerable number of the articles published from 2013 to date were regarding treatment and diagnostics (Figure 6). Under the umbrella of therapeutic articles, compared to top 100 articles, the work on surgical management has increased considerably especially on the comparison of laparoscopic procedures to open gastric resection (Figure 7). We also noticed that researchers have now been trying new medicinal therapies for GISTs, like olaratumab, regorafenib and other recent drugs, in addition to previous ones like sunitinib and imatinib. Although work on diagnostics of GISTs has increased over time and most studied topics were radiographic and needle aspiration techniques, it is an understudied area warranting further work. Along with these, basic sciences were also kept under focus by scientists; the subgroup of genetics, especially, has been studied vigorously in past five years. A number of recent studies were extensive enough to add literature under the umbrella of multiple subjects, the majority of which contributed to the arena of genetics and medicinal therapy. Moreover, in accordance with the top 100 articles, the latest top cited articles were also mostly contributed by the USA and Harvard Medical School.

Although efforts were made to eliminate any bias, certain limitations must be considered. Firstly, a major limitation was possible citation bias, including in-house citations, negative citations and incomplete citations. Secondly, only one database was used to extract the list which may have resulted in overlooking of some articles that were not recognized by Scopus. Scopus has been reported to miss older citations, which results in the omission of researches conducted and published prior to 1980 [27,28]. Our list may have missed some Citation Classics, it can be explained as ‘obliteration by incorporation’ [17], which in simple terms states that the content of some classic articles has become such common knowledge that they no longer require citation.

## Conclusions

A bibliometric analysis on GIST helped in identifying it as an escalating topic of discussion. The last two decades have shown a significant increase in the relevant GIST studies in comparison to the scarce and wide-spread publications in the entire twentieth century. Citation count of the articles remained uninfluenced by the journal’s Citescore and the article type. Areas pertaining to basic sciences and genetics have shown great progress whereas surgical management and advances in diagnostic aspects of GIST have yet to reach the same level of success.

The prevalence of GIST is highest in China, regardless of which, the country and institution with the highest number of citation contribution are the USA and Harvard Medical College, respectively. Furthermore, our analysis suggests that greater amount of time and attention towards research focusing on advances in diagnostic investigations, surgical treatment, and target therapy is required in order to improve the overall prognosis of the disease.

## Appendices

Appendix A

**Table 5:** Top 100 articles on gastrointestinal stromal tumors (GISTs).

Rank	Article	Total Citation	Citation Density	Most Cited in (year)
1	Hirota S, Isozaki K, Moriyama Y, et al.: Gain-of-function mutations of c-kit in human gastrointestinal stromal tumors. Science 1998, 279:577-580.	3,076	161.895	2006
2	Demetri GD, Von Mehren M, Blanke CD, et al.: Efficacy and safety of imatinib mesylate in advanced gastrointestinal stromal tumors. N Engl J Med 2002, 347:472-480.	3,047	203.133	2006
3	Fletcher CD, Berman JJ, Corless C, et al.: Diagnosis of gastrointestinal stromal tumors: A consensus approach. Hum Pathol 2002, 33:459-465.	2,278	151.867	2009
4	DeMatteo RP, Lewis JJ, Leung D, et al.: Two hundred gastrointestinal stromal tumors: Recurrence patterns and prognostic factors for survival. Ann Surg 2000, 231:251.	1,746	102.706	2006
5	Joensuu H, Roberts PJ, Sarlomo-Rikala M, et al.: Effect of the tyrosine kinase inhibitor sti571 in a patient with a metastatic gastrointestinal stromal tumor. N Engl J Med 2001, 344:1052-1056.	1,601	100.663	2003
6	Heinrich MC, Corless CL, Demetri GD, et al.: Kinase mutations and imatinib response in patients with metastatic gastrointestinal stromal tumor. J Clin Oncol 2003, 21:4342-4349.	1,599	114.214	2006
7	Demetri GD, van Oosterom AT, Garrett CR, et al.: Efficacy and safety of sunitinib in patients with advanced gastrointestinal stromal tumour after failure of imatinib: A randomised controlled trial. Lancet 2006, 368:1329-1338.	1,571	142.818	2009
8	Heinrich MC, Corless CL, Duensing A, et al.: Pdgfra activating mutations in gastrointestinal stromal tumors. Science 2003, 299:708-710.	1,561	111.5	2010
9	Wahl RL, Jacene H, Kasamon Y, et al.: From recist to percist: Evolving considerations for pet response criteria in solid tumors. J Nucl Med 2009, 50:122S-150S.	1,340	167.5	2015
10	Miettinen M, Lasota J. Gastrointestinal stromal tumors-definition, clinical, histological, immunohistochemical, and molecular genetic features and differential diagnosis. Virchows Arch 2001, 438:1-12.	1,265	79.063	2005
11	Kindblom L-G, Remotti HE, Aldenborg F, et al.: Gastrointestinal pacemaker cell tumor (gipact): Gastrointestinal stromal tumors show phenotypic characteristics of the interstitial cells of cajal. Am J Pathol 1998, 152:1259.	1,262	66.421	2002
12	Verweij J, Casali PG, Zalcberg J, et al.: Progression-free survival in gastrointestinal stromal tumours with high-dose imatinib: Randomised trial. Lancet 2004, 364:1127-1134.	1,146	88.154	2008
13	van Oosterom AT, Judson I, Verweij J, et al.: Safety and efficacy of imatinib (sti571) in metastatic gastrointestinal stromal tumours: A phase i study. Lancet 2001, 358:1421-1423.	1,109	69.313	2003
14	Miettinen M, Lasota J. Gastrointestinal stromal tumors: Pathology and prognosis at different sites. Semin Diagn Pathol 2006, 23:70-83.	848	94.222	2015
15	Corless CL, Fletcher JA, Heinrich MC. Biology of gastrointestinal stromal tumors. J Clin Oncol 2004, 22:3813-3825.	834	64.154	2008
16	Choi H, Charnsangavej C, Faria SC, et al.: Correlation of computed tomography and positron emission tomography in patients with metastatic gastrointestinal stromal tumor treated at a single institution with imatinib mesylate: Proposal of new computed tomography response criteria. J Clin Oncol 2007, 25:1753-1759.	814	81.4	2014
17	Miettinen M, Lasota J. Gastrointestinal stromal tumors: Review on morphology, molecular pathology, prognosis, and differential diagnosis. Arch Pathol Lab Med 2006, 130:1466-1478.	787	71.546	2010
18	Miettinen M, Sobin LH, Lasota J. Gastrointestinal stromal tumors of the stomach: A clinicopathologic, immunohistochemical, and molecular genetic study of 1765 cases with long-term follow-up. Am J Surg Pathol 2005, 29:52-68.	787	65.583	2009
19	Rubin BP, Singer S, Tsao C, et al.: Kit activation is a ubiquitous feature of gastrointestinal stromal tumors. Cancer Res 2001, 61:8118-8121.	781	48.813	2003
20	Jain RK, Duda DG, Clark JW, et al.: Lessons from phase iii clinical trials on anti-vegf therapy for cancer. Nat Clin Pract Oncol 2006, 3:24-40.	764	69.455	2007
21	Nilsson B, Bümming P, Meis‐Kindblom JM, et al.: Gastrointestinal stromal tumors: The incidence, prevalence, clinical course, and prognostication in the preimatinib mesylate era. Cancer 2005, 103:821-829.	754	62.833	2015
22	DeMatteo RP, Ballman KV, Antonescu CR, et al.: Adjuvant imatinib mesylate after resection of localised, primary gastrointestinal stromal tumour: A randomised, double-blind, placebo-controlled trial. Lancet 2009, 373:1097-1104.	738	123	2012
23	Miettinen M, Sarlomo-Rikala M, Lasota J. Gastrointestinal stromal tumors: Recent advances in understanding of their biology. Hum Pathol 1999, 30:1213-1220.	665	36.944	2003
24	Sarlomo-Rikala M, Kovatich AJ, Barusevicius A, et al.: CD117: A sensitive marker for gastrointestinal stromal tumors that is more specific than CD34. Mod Pathol 1998,11:728-734.	641	33.737	2002
25	Chow LQ, Eckhardt SG. Sunitinib: From rational design to clinical efficacy. J Clin Oncol 2007, 25:884-896.	614	61.4	2012
26	Blanke CD, Rankin C, Demetri GD, et al.: Phase iii randomized, intergroup trial assessing imatinib mesylate at two dose levels in patients with unresectable or metastatic gastrointestinal stromal tumors expressing the kit receptor tyrosine kinase: S0033. J Clin Oncol 2008, 26:626-632.	605	67.222	2012
27	Debiec-Rychter M, Sciot R, Le Cesne A, et al.: Kit mutations and dose selection for imatinib in patients with advanced gastrointestinal stromal tumours. Eur J Cancer 2006, 42:1093-1103.	594	54	2012
28	Blanke CD, Demetri GD, Von Mehren M, et al.: Long-term results from a randomized phase ii trial of standard-versus higher-dose imatinib mesylate for patients with unresectable or metastatic gastrointestinal stromal tumors expressing kit. J Clin Oncol 2008, 26:620-625.	564	62.667	2012
29	Tuveson DA, Willis NA, Jacks T, et al.: Sti571 inactivation of the gastrointestinal stromal tumor c-kit oncoprotein: Biological and clinical implications. Oncogene 2001, 20:5054.	533	33.313	2003
30	Lux ML, Rubin BP, Biase TL, et al.: Kit extracellular and kinase domain mutations in gastrointestinal stromal tumors. Am J Pathol 2000, 156:791-795.	525	30.882	2003
31	Heinrich MC, Blanke CD, Druker BJ, Corless CL: Inhibition of kit tyrosine kinase activity: A novel molecular approach to the treatment of kit-positive malignancies. J Clin Oncol 2002, 20:1692-1703.	518	34.533	2005
32	Heinrich MC, Corless CL, Blanke CD, et al.: Molecular correlates of imatinib resistance in gastrointestinal stromal tumors. J Clin Oncol 2006, 24:4764-4774.	517	47	2009
33	Antonescu CR, Besmer P, Guo T, et al.: Acquired resistance to imatinib in gastrointestinal stromal tumor occurs through secondary gene mutation. Clin Cancer Res 2005, 11:4182-4190.	515	42.917	2011
34	Miettinen M, Leslie H, Lasota J: Evaluation of malignancy and prognosis of gastrointestinal stromal tumors: a review. Hum pathol 2002, 33:478-483.	514	34.267	2005
35	Hirota S, Ohashi A, Nishida T, Isozaki K, Kinoshita K, Shinomura Y, Kitamura Y: Gain-of-function mutations of platelet-derived growth factor receptor α gene in gastrointestinal stromal tumors. Gastroenterol 2003, 125:660-667.	512	36.571	2008
36	Lasota J, Jasinski M, Sarlomo-Rikala M, Miettinen M: Mutations in exon 11 of c-Kit occur preferentially in malignant versus benign gastrointestinal stromal tumors and do not occur in leiomyomas or leiomyosarcomas. Am J Pathol 1999, 154:53-60.	495	27.5	2002
37	Dematteo RP, Heinrich MC, Wa'el M ER, Demetri G: Clinical management of gastrointestinal stromal tumors: before and after STI-571. Hum Pathol 2002, 33:466-477.	487	32.467	2005
38	Corless CL, Schroeder A, Griffith D, et al.: PDGFRA mutations in gastrointestinal stromal tumors: frequency, spectrum and in vitro sensitivity to imatinib. J Clin Oncol 2005, 23:5357-5364.	483	40.25	2008
39	Taniguchi M, Nishida T, Hirota S, et al.: Effect of c-kit mutation on prognosis of gastrointestinal stromal tumors. Cancer Res 1999, 59:4297-4300.	474	26.333	2003
40	Miettinen M, Virolainen M: Gastrointestinal stromal tumors-value of CD34 antigen in their identification and separation from true leiomyomas and schwannomas. Am J Surg Pathol 1995, 19:207-216.	470	21.364	2001
41	Nishida T, Hirota S, Taniguchi M, et al.: Familial gastrointestinal stromal tumours with germline mutation of the KIT gene. Nat Genet 1998, 19:323-324.	448	23.579	2002
42	Sircar K, Hewlett BR, Huizinga JD, Chorneyko K, Berezin I, Riddell RH: Interstitial cells of Cajal as precursors of gastrointestinal stromal tumors. Am J Surg Pathol 1999, 23:377-389.	446	24.778	2002
43	Blay JY, Bonvalot S, Casali P, et al.: Consensus meeting for the management of gastrointestinal stromal tumors Report of the GIST Consensus Conference of 20–21 March 2004, under the auspices of ESMO. Ann Oncol 2005, 16:566-578.	443	36.91	2009
44	Joensuu H, Fletcher C, Dimitrijevic S ,et al.: Management of malignant gastrointestinal stromal tumours. Lancet Oncol 2002, 3:655-664.	441	29.4	2006
45	Nielsen TO, West RB, Linn SC, et al.: Molecular characterisation of soft tissue tumours: a gene expression study. Lancet 2002, 359:1301-1307.	436	29.067	2006
46	Pidhorecky I, Cheney RT, Kraybill WG, Gibbs JF: Gastrointestinal stromal tumors: current diagnosis, biologic behavior, and management. Ann Surg Oncol 2000, 7:705-712.	426	25.059	2006
47	Stroobants S, Goeminne J, Seegers M, et al.: 18 FDG-positron emission tomography for the early prediction of response in advanced soft tissue sarcoma treated with imatinib mesylate (Glivec®). Eur J Cancer 2003, 39:2012-2020.	420	30	2006;2008
48	Miettinen M, Sobin LH, Sarlomo-Rikala M: Immunohistochemical spectrum of GISTs at different sites and their differential diagnosis with a reference to CD117 (KIT). Mod Pathol 2000, 13:1134.	419	24.647	2005
49	Miettinen M, Monihan JM, Sarlomo-Rikala M, Kovatich AJ, Carr NJ, Emory TS, Sobin LH: Gastrointestinal stromal tumors/smooth muscle tumors (GISTs) primary in the omentum and mesentery: clinicopathologic and immunohistochemical study of 26 cases. Am J Surg Pathol 1999, 23:1109.	417	23.167	2003
50	Force NT, Demetri G, Benjamin R, et al.: Nccn task force report: Management of patients with gastrointestinal stromal tumor (gist): Update of the nccn clinical practice guidelines. J Natl Compr Canc Netw 2007, 5:S1-S29.	398	56.857	2015
51	Demetri GD, Von Mehren M, Antonescu CR, et al.: Nccn task force report: Update on the management of patients with gastrointestinal stromal tumors. J Natl Compr Canc Netw 2010, 8:S-1-S-41.	394	39.4	2009
52	Heinrich MC, Rubin BP, Longley BJ, Fletcher JA: Biology and genetic aspects of gastrointestinal stromal tumors: KIT activation and cytogenetic alterations. Hum Pathol 2002, 33:484-495.	390	26	2003;2005
53	Miettinen M, Makhlouf H, Sobin LH, Lasota J: Gastrointestinal stromal tumors of the jejunum and ileum: a clinicopathologic, immunohistochemical, and molecular genetic study of 906 cases before imatinib with long-term follow-up. Am J Surg Pathol 2006, 30:477-489.	385	35	2010
54	Joensuu H, Eriksson M, Hall KS, et al.: One vs three years of adjuvant imatinib for operable gastrointestinal stromal tumor: a randomized trial. Jama 2012, 307:1265-1272.	383	76.6	2013
55	Benjamin RS, Choi H, Macapinlac HA, et al.: We should desist using RECIST, at least in GIST. J Clin Oncol 2007, 25:1760-1764.	380	38	2010
56	Joensuu H: Risk stratification of patients diagnosed with gastrointestinal stromal tumor. Hum Pathol 2008, 39:1411-1419.	379	42.111	2015
57	Heinrich MC, Maki RG, Corless CL, et al.: Primary and secondary kinase genotypes correlate with the biological and clinical activity of sunitinib in imatinib-resistant gastrointestinal stromal tumor. J Clin Oncol 2008, 26:5352-5359.	376	41.778	2009;2010
58	Shawver LK, Slamon D, Ullrich A: Smart drugs: tyrosine kinase inhibitors in cancer therapy. Cancer cell 2002, 1:117-123.	374	24.933	2003
59	West RB, Corless CL, Chen X, et al.: The novel marker, DOG1, is expressed ubiquitously in gastrointestinal stromal tumors irrespective of KIT or PDGFRA mutation status. Am J Pathol 2004, 165:107-113.	373	28.692	2015
60	Debiec-Rychter M, Cools J, Dumez H, et al.: Mechanisms of resistance to imatinib mesylate in gastrointestinal stromal tumors and activity of the PKC412 inhibitor against imatinib-resistant mutants. Gastroenterol 2005, 128:270-279.	369	30.75	2007
61	Demetri GD, Reichardt P, Kang YK, et al.: Efficacy and safety of regorafenib for advanced gastrointestinal stromal tumours after failure of imatinib and sunitinib (GRID): an international, multicentre, randomised, placebo-controlled, phase 3 trial. Lancet 2013, 381:295-302.	368	92	2015
62	Corless CL, McGreevey L, Haley A, Town A, Heinrich MC: KIT mutations are common in incidental gastrointestinal stromal tumors one centimeter or less in size. Am J Pathol 2002, 160:1567-1572.	365	24.333	2004;2005
63	Ma Y, Zeng S, Metcalfe DD, et al.: The c-KIT mutation causing human mastocytosis is resistant to STI571 and other KIT kinase inhibitors; kinases with enzymatic site mutations show different inhibitor sensitivity profiles than wild-type kinases and those with regulatory-type mutations. Blood 2002, 99:1741-1744.	365	24.333	2004
64	Miettinen M, Majidi M, Lasota J: Pathology and diagnostic criteria of gastrointestinal stromal tumors (GISTs): a review Eur J Cancer 2002, 38:S39-51.	362	24.133	2007
65	Verweij J, van Oosterom A, Blay JY, et al.: Imatinib mesylate (STI-571 Glivec®, Gleevec™) is an active agent for gastrointestinal stromal tumours, but does not yield responses in other soft-tissue sarcomas that are unselected for a molecular target: results from an EORTC Soft Tissue and Bone Sarcoma Group phase II study. Eur J Cancer 2003, 39:2006-2011.	355	25.357	2005
66	Singer S, Rubin BP, Lux ML, Chen CJ, Demetri GD, Fletcher CD, Fletcher JA: Prognostic value of KIT mutation type, mitotic activity, and histologic subtype in gastrointestinal stromal tumors. J Clin Oncol 2002, 20:3898-3905.	355	23.667	2005
67	Rubin BP, Heinrich MC, Corless CL: Gastrointestinal stromal tumour. Lancet 2007, 369:1731-1741.	348	34.8	2006
68	Debiec-Rychter M, Dumez H, Judson I, et al.: Use of c-KIT/PDGFRA mutational analysis to predict the clinical response to imatinib in patients with advanced gastrointestinal stromal tumours entered on phase I and II studies of the EORTC Soft Tissue and Bone Sarcoma Group. Eur J Cancer 2004, 40:689-695.	348	26.769	2008
69	Miettinen M, Furlong M, Sarlomo-Rikala M, Burke A, Sobin LH, Lasota J: Gastrointestinal stromal tumors, intramural leiomyomas, and leiomyosarcomas in the rectum and anus: a clinicopathologic, immunohistochemical, and molecular genetic study of 144 cases. Am J Surg Pathol 2001, 25:1121-1133.	348	21.75	2008
70	Medeiros F, Corless CL, Duensing A, et al.: KIT-negative gastrointestinal stromal tumors: proof of concept and therapeutic implications. Am J Surg Pathol 2004, 28:889-894.	343	26.385	2006
71	Peng B, Lloyd P, Schran H: Clinical pharmacokinetics of imatinib. Clin Pharmacokinet 2005, 44:879-894.	338	28.167	2010
72	Tran T, Davila JA, El-Serag HB: The epidemiology of malignant gastrointestinal stromal tumors: an analysis of 1,458 cases from 1992 to 2000. Am J Gastroenterol 2005, 100:162.	337	28.083	2009
73	Heinrich MC, Owzar K, Corless CL, et al.: Correlation of kinase genotype and clinical outcome in the North American Intergroup Phase III Trial of imatinib mesylate for treatment of advanced gastrointestinal stromal tumor: CALGB 150105 Study by Cancer and Leukemia Group B and Southwest Oncology Group. J Clin Oncol 2008, 26:5360-367.	335	37.222	2012
74	Reith JD, Goldblum JR, Lyles RH, Weiss SW: Extragastrointestinal (soft tissue) stromal tumors: an analysis of 48 cases with emphasis on histologic predictors of outcome. Mod Pathol 2000, 13:577.	334	19.647	2011
75	Cook JR, Dehner LP, Collins MH, Ma Z, Morris SW, Coffin CM, Hill DA: Anaplastic lymphoma kinase (ALK) expression in the inflammatory myofibroblastic tumor: a comparative immunohistochemical study. Am J Surg Pathol 2001, 25:1364-1371.	333	20.813	2015
76	Dagher, R. Cohen, M. Williams, et al.: Approval summary: Imatinib mesylate in the treatment of metastatic and/or unresectable malignant gastrointestinal stromal tumors. Clin Cancer Res 2002, 10:3034-3038	330	19.412	2007
77	Miettinen M, Sarlomo-Rikala M, Sobin LH, Lasota J: Esophageal stromal tumors: a clinicopathologic, immunohistochemical, and molecular genetic study of 17 cases and comparison with esophageal leiomyomas and leiomyosarcomas. Am J Surg Pathol 2000; 24:211-222.	314	18.471	2006
78	Therasse P, Eisenhauer EA, Verweij J: RECIST revisited: A review of validation studies on tumour assessment. Eur J Cancer 2006, 42:1031-1039.	313	28.455	2009
79	Choi H, Charnsangavej C, Faria SD, et al.: CT evaluation of the response of gastrointestinal stromal tumors after imatinib mesylate treatment: a quantitative analysis correlated with FDG PET findings AJR Am J Roentgenol 2004, 183:1619-1628.	310	23.846	2012
80	Levy AD, Remotti HE, Thompson WM, Sobin LH, Miettinen M: From the archives of the AFIP: gastrointestinal stromal tumors: radiologic features with pathologic correlation. Radiographics 2003, 23:283-304.	303	21.643	2001
81	Ueyama T, Guo KJ, Hashimoto H, Daimaru Y, Enjoji M: A clinicopathologic and immunohistochemical study of gastrointestinal stromal tumors. Cancer 1992, 69:947-955.	303	12.12	2012
82	Miettinen M, Kopczynski J, Makhlouf HR, et al.: Gastrointestinal stromal tumors, intramural leiomyomas, and leiomyosarcomas in the duodenum: a clinicopathologic, immunohistochemical, and molecular genetic study of 167 cases. Am J Surg Pathol 2003, 27:625-641.	298	21.286	2012
83	Goodman VL, Rock EP, Dagher R, et al.: Approval summary: sunitinib for the treatment of imatinib refractory or intolerant gastrointestinal stromal tumors and advanced renal cell carcinoma. Clin Cancer Res 2007, 13:1367-1373.	296	29.6	2009
84	Burkill GJ, Badran M, Al-Muderis O, Meirion Thomas J, Judson IR, Fisher C, Moskovic EC: Malignant gastrointestinal stromal tumor: distribution, imaging features, and pattern of metastatic spread. Radiology. 2003, 226:527-532.	296	21.143	2011
85	Franquemont DW: Differentiation and risk assessment of gastrointestinal stromal tumors. Am J Clin Pathol 1995, 103:41-47.	293	13.318	2001
86	Ando N, Goto H, Niwa Y, Hirooka Y, Ohmiya N, Nagasaka T, Hayakawa T: The diagnosis of GI stromal tumors with EUS-guided fine needle aspiration with immunohistochemical analysis. Gastrointest Endosc 2002, 55:37-43.	289	19.267	2005;2009
87	DeMatteo RP, Gold JS, Saran L, et al.: Tumor mitotic rate, size, and location independently predict recurrence after resection of primary gastrointestinal stromal tumor (GIST). Cancer 2008, 112:608-615.	287	31.889	2012
88	Lasota J, Wozniak A, Sarlomo-Rikala M, et al.: Mutations in exons 9 and 13 of KIT gene are rare events in gastrointestinal stromal tumors: a study of 200 cases. Am J Pathol 2000, 157:1091-1095	285	16.765	2003
89	Blay JY, Le Cesne A, Ray-Coquard I, et al.: Prospective multicentric randomized phase III study of imatinib in patients with advanced gastrointestinal stromal tumors comparing interruption versus continuation of treatment beyond 1 year: the French Sarcoma Group. J Clin Oncol 2007, 25:1107-1113.	281	28.1	2009
90	Corless CL, Barnett CM, Heinrich MC: Gastrointestinal stromal tumours: origin and molecular oncology. Nature reviews. Cancer 2011, 11:865.	280	46.667	2013
91	Raut CP, Posner M, Desai J, et al.: Surgical management of advanced gastrointestinal stromal tumors after treatment with targeted systemic therapy using kinase inhibitors. J Clin Oncol 2006, 24:2325-2331.	277	25.128	2012
92	Antonescu CR, Sommer G, Sarran L, et al,: Association of KIT exon 9 mutations with nongastric primary site and aggressive behavior. Clin Cancer Res 2003, 9:3329-3337.	274	19.571	2003
93	Pierie JP, Choudry U, Muzikansky A, Yeap BY, Souba WW, Ott MJ: The effect of surgery and grade on outcome of gastrointestinal stromal tumors. Arch Surg 2001, 136:383-389.	274	17.125	2006;2008;2012
94	Desai J, Yassa L, Marqusee E, et al.: Hypothyroidism after sunitinib treatment for patients with gastrointestinal stromal tumors. Ann Intern Med 2006, 145:660-664.	273	24.818	2007
95	Chen LL, Trent JC, Wu EF, et al.: A missense mutation in KIT kinase domain 1 correlates with imatinib resistance in gastrointestinal stromal tumors. Cancer Res 2004, 64:5913-5919.	267	20.538	2004;2005
96	Janeway KA, Kim SY, Lodish M, et al.: Defects in succinate dehydrogenase in gastrointestinal stromal tumors lacking KIT and PDGFRA mutations. Proc Natl Acad Sci U S A. 2011, 108:314-318.	266	44.333	2008
97	Zalcberg JR, Verweij J, Casali PG, et al.: Outcome of patients with advanced gastro-intestinal stromal tumours crossing over to a daily imatinib dose of 800mg after progression on 400mg. Eur J Cancer 2005, 41:1751-1757.	266	22.167	2013
98	Joensuu H, Vehtari A, Riihimäki J, et al.: Risk of recurrence of gastrointestinal stromal tumour after surgery: an analysis of pooled population-based cohorts. Lancet Oncol 2012, 13:265-274.	261	52.2	2015
99	Novitsky YW, Kercher KW, Sing RF, Heniford BT: Long-term outcomes of laparoscopic resection of gastric gastrointestinal stromal tumors. Ann Surg 2006, 243:738.	260	23.636	2006
100	Tamborini E, Bonadiman L, Greco A, et al.: A new mutation in the KIT ATP pocket causes acquired resistance to imatinib in a gastrointestinal stromal tumor patient. Gastroenterology 2004, 127:294-299.	260	20	2012

## References

[1] Regehr G (2004). Trends in medical education research. Acad Med.

[2] Sackett DL (1997). Evidence-based medicine. Semin Perinatol.

[3] Benton DC (2017). Using bibliometrics to support revalidation requirements. Nurs Stand.

[4] Moed HF (2002). The impact-factors debate: the ISI's uses and limits. Nature.

[5] Pendlebury DA (2010). White paper using bibliometrics: a guide to evaluating research performance with citation data. Thomson Reuters.

[6] Glynn RW, Scutaru C, Kerin MJ, Sweeny KJ (2010). Breast cancer research output, 1945-2008: a bibliometric and density-equalizing analysis. Breast Cancer Res.

[7] Kelly J, Glynn R, O'Briain D, Felle P, McCabe JP (2010). The 100 classic papers of orthopaedic surgery: a bibliometric analysis. J Bone Joint Surg Br.

[8] Ibrahim GM, Carter Snead O, Rutka JT, Lozano AM (2012). The most cited works in epilepsy: trends in the “citation classics”. Epilepsia.

[9] Siddiqi TJ, Usman MS, Khan MS (2017). The 100 most influential papers in the field of thrombolytic therapy: a bibliometric analysis. Am J Cardiovasc Drugs.

[10] Usman MS, Siddiqi TJ, Khan MS, Fatima K, Butler J, Manning WJ, Khosa F (2017). A scientific analysis of the 100 citation classics of valvular heart disease. Am J Cardiol.

[11] Rubin BP, Heinrich MC, Corless CL (2007). Gastrointestinal stromal tumour. Lancet.

[12] Søreide K, Sandvik OM, Søreide JA, Giljasa V, Jureckova A, Bulusu VR (2016). Global epidemiology of gastrointestinal stromal tumours (GIST): a systematic review of population-based cohort studies. Cancer Epidemiol.

[13] DeVita VT, Lawrence TS, Rosenberg SA (2008). DeVita, Hellman, and Rosenberg's Cancer: Principles and Practice of Oncology. https://oncouasd.files.wordpress.com/2014/09/cancer-principles-and-practice-of-oncology-6e.pdf.

[14] Kingham TP, DeMatteo RP (2009). Multidisciplinary treatment of gastrointestinal stromal tumors. Surg Clin North Am.

[15] Chaudhry UI, DeMatteo RP (2009). Management of resectable gastrointestinal stromal tumor. Hematol Oncol Clin North Am.

[16] Gold JS, DeMatteo RP (2007). Neoadjuvant therapy for gastrointestinal stromal tumor (GIST): racing against resistance. Ann Surg Oncol.

[17] Garfield E (1987). 100 citation classics from the Journal of the American Medical Association. Jama.

[18] Falagas ME, Pitsouni EI, Malietzis GA, Paappas G (2008). Comparison of PubMed, Scopus, Web of Science, and Google Scholar: strengths and weaknesses. FASEB J.

[19] Kindblom LG, Remotti HE, Aldenborg F, Meis-Kindblom JM (1998). Gastrointestinal pacemaker cell tumor (GIPACT): gastrointestinal stromal tumors show phenotypic characteristics of the interstitial cells of Cajal. Am J Pathol.

[20] Rubin BP (2001). Recent progress in the classification of soft tissue tumors: role of genetics and clinical implications. Curr Opin Oncol.

[21] Zijlstra H, McCullough R (2016). CiteScore: a new metric to help you track journal performance and make decisions. Elsevier.

[22] Paladugu R, Schein M, Gardezi S, Wise L (2002). One hundred citation classics in general surgical journals. World J Surg.

[23] Campbell FM (1990). National bias a comparison of citation practices by health professionals. Bull Med Libr Assoc.

[24] Stossel TP (1987). Volume: papers and academic promotion. Ann Intern Med.

[25] Nash-Stewart CE (2012). Does Bradford’s law of scattering predict the size of the literature in cochrane reviews?. J Med Libr Assoc.

[26] La Torre G, Sciarra I, Chiappetta M, Monteduro A (2017). New bibliometric indicators for the scientific literature: an evolving panorama. (Article in Italian). Clin Ter.

[27] Brandt JS, Downing AC, Howard DL, Kofinas JD, Chasen ST (2010). Citation classics in obstetrics and gynecology: the 100 most frequently cited journal articles in the last 50 years. Am J Obstet Gynecol.

[28] Bakkalbasi N, Bauer K, Glover J, Wang L (2006). Three options for citation tracking: Google Scholar, Scopus and Web of Science. Biomed Digit Libr.

